# Herpesvirus and Subsequent Usutu Virus Infection in a Great Grey Owl (*Strix nebulosa*) at the Ljubljana Zoo, Slovenia

**DOI:** 10.3390/ani14081200

**Published:** 2024-04-17

**Authors:** Zoran Žlabravec, Pavel Kvapil, Brigita Slavec, Olga Zorman Rojs, Tanja Švara, Jožko Račnik

**Affiliations:** 1 Institute for Poultry, Birds, Small Mammals, and Reptiles, Veterinary Faculty, University of Ljubljana, Gerbičeva ulica 60, 1000 Ljubljana, Slovenia; zoran.zlabravec@vf.uni-lj.si (Z.Ž.);; 2 Veterinary Department, Ljubljana Zoo, Večna Pot 70, 1000 Ljubljana, Slovenia; 3 Institute of Pathology, Wild Animals, Fish and Bees, Veterinary Faculty, University of Ljubljana, Gerbičeva ulica 60, 1000 Ljubljana, Slovenia; tanja.svara@vf.uni-lj.si

**Keywords:** herpesvirus, Usutu virus, great grey owl, PCR, RT-PCR, zoo, Slovenia

## Abstract

**Simple Summary:**

This case report describes the detection and continuous shedding of herpesvirus (HV) and subsequent Usutu virus (USUV) infection in a great grey owl at the Ljubljana Zoo. Mild abnormalities were detected through diagnostic procedures; however, no obviously clinical signs related to HV disease were detected. Because of occasionally described high rates of lethal HV disease, the poor response to previous treatment, and the potential source of infection for other birds and animals at the zoo, therapy with an antiviral drug was performed and led to the termination of HV shedding. During the period of no detected HV and no clinical signs of illness, the owl suddenly died because of USUV infection. To the best of our knowledge, this is the first report of HV and the presence of USUV in a great grey owl in Slovenia.

**Abstract:**

Herpesvirus (HV) has been known to cause disease in owls, with various clinical signs and outcomes for the last several decades. The HV *DNA polymerase* gene was detected in oropharyngeal and cloacal swabs of a male great grey owl (*Strix nebulosa*) in a zoological collection in Ljubljana, Slovenia. In the following 4 months, despite continuous HV detection in swabs, no clinical signs with a clear link to HV disease were observed. Hepatoprotective and immunostimulant therapies applied during this period did not prevent HV shedding. Therefore, peroral antiviral therapy with acyclovir (150 mg/kg q24 h for seven days) was performed, and the owl tested negative at the next sampling and remained negative for the next 8 months. After that, the owl again tested positive for HV presence, and the same protocol with antiviral therapy was performed. After 3 weeks with a negative test for HV presence, without any clinical signs of illness, the owl suddenly died because of Usutu virus (USUV) infection. Among all the owls at the zoo, interestingly, only the HV-positive great grey owl died because of USUV infection. The USUV sequence detected and obtained in this study clusters together with other Europe 2 sequences detected in neighboring countries. Our study shows the potential of acyclovir therapy in the prevention of herpesvirus shedding and, moreover, lowering the possibility for spreading HV to other owls and birds. To the best of our knowledge, this is the first report of HV presence and USUV infection in a great grey owl in Slovenia.

## 1. Introduction

Herpesviruses (HVs) are double-stranded DNA viruses known to infect a wide range of vertebrates, including birds, mammals, amphibians, reptiles, and fish [1]. In free-living and captive owls (Strigiformes), HV causes a highly lethal disease known as inclusion body disease or herpesvirus hepatitis. The disease severity depends on the host’s immune status, the host–virus relationship, and the presence of a secondary infection [2]. HVs have the ability to establish lifelong latency within the host and to periodically reactivate, and it is not unusual that an individual might be carrying the pathogen without any clinical signs of disease until exposure to an environmental stressor triggers the activation of viral replication and spread to a new host organism [2]. Generally, affected owls are depressed and anorectic from 2 to 5 days before death occurs. Leukopenia may be noted during the acute phase of the infection [3]. Necrosis of the liver, spleen, and bone marrow, with intranuclear eosinophilic inclusion bodies in the cells of these organs is characteristic for HV-infected birds, and in some owls, yellow nodules have been observed in the buccal palate and esophagus [2]. Keratitis and conjunctivitis may develop in surviving owls [4]. However, in recent research, HV has also been detected in owls without any clinical or productivity (i.e., clutch or brood size) deviations [5].

The disease has been reported in the great horned owl (*Bubo virginianus*) [4], Eurasian eagle owl (*Bubo bubo*), long-eared owl (*Asio otus*), snowy owl (*Bubo scandiacus*), little owl (*Athene noctua*), and boreal owl (*Aegolius funereus*) [6,7]. Numerous field studies and the detection of HV, endemic in the pigeon population—namely, *Mardivirus columbid alpha 1* (CoAHV-1) from the genus *Mardivirus*, subfamily *Alphaherpesvirinae*—in owls and other raptors suggest that the consumption of infected pigeons is the most likely mode of HV infection in hawks, eagles, and owls [8,9,10]. However, recent studies have shown that other HVs that are different from CoAHV-1 are also present in owl species [4,11].

Usutu virus (USUV) is a mosquito-borne flavivirus and a member of the Japanese encephalitis virus group. This virus is primarily transmitted through a vertebrate host (predominantly birds) through the mosquito’s life cycle [12]. In Europe, USUV has been shown to be highly pathogenic for several bird species, especially blackbirds (*Turdus merula*), great grey owls, and house sparrows (*Passer domesticus*) [13,14,15]. USUV has wide tropism and virulence in a variety of tissues and organs. The most frequently reported clinical signs are prostration, disorientation, ataxia, and weight loss. Hepatomegaly and splenomegaly are the main macroscopic lesions. Necrotic areas and inflammatory infiltrates composed of lymphoid and histiocytic cells have also been reported in the heart, liver, kidneys, spleen, and brains of infected birds. Glial nodules and neuronophagia have also been observed in the brain [13,16].

The present case features long-term presence, shedding, and therapy for HV infection and, subsequently, USUV infection in a captive great grey owl at the Ljubljana Zoo in Slovenia.

## 2. Case Description

On 18 December 2020, an 18-month-old male great grey owl (*Strix nebulosa*) arrived at the Ljubljana Zoo in Slovenia from the Ranua Resort wildlife park in Finland. During the clinical exam, the owl had a 3.5/5 body condition score (BCS), weighed 1000 g (normal weight), and was bright, active, and alert and able to perch normally. No obvious abnormalities were observed during the external physical examination. On 11 January 2021, the owl weighed 1123 g, and the BCS was 3.5/5. Oropharyngeal and cloacal swabs tested negative for avian influenza (AI), avian paramyxovirus (PMV), *Chlamydia*, West Nile virus (WNV), and USUV using standard molecular diagnostic methods [17,18,19,20,21]. The owl was vaccinated against WNV (Proteq West Nile, Merial, Bohringer-Ingelheim, Lyon, France). After 6 weeks of quarantine, on 30 January 2021, because of improper handling by a caretaker, the great grey owl escaped from the aviary enclosure to the nearby forest and was missing for 1 month. After it was caught on 28 February 2021, the owl weighed 990 g. The results of the microscopic fecal examination showed the presence of *Capillaria* spp., Strongylidae, and *Eimeria* spp. Treatment with marbofloxacin (Marfloxin, Krka, Novo Mesto, Slovenia), meloxicam (Meloxidolor, Le Vet. Beheer, Utrecht, The Netherlands), fenbendazole (Panacur, Intervet Int., MSD, Boxmeer, The Netherlands), sulfamethoxazole trimethoprim (Trisulfon, Krka, Novo Mesto, Slovenia), and subcutaneous Ringer’s solution with Duphalyte (Zoetis, Madrid, Spain) was administered. The bird was revaccinated against WNV.

**Figure 1:**
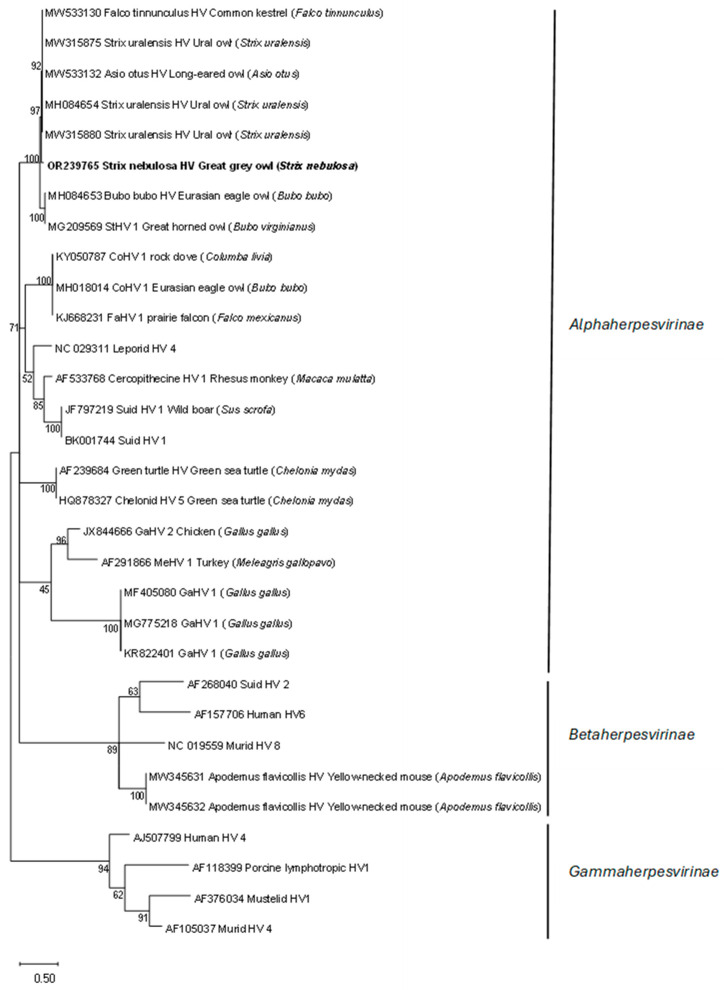
Phylogenetic relationship based on partial *DNA polymerase* gene nucleotide sequences of HV detected in the great grey owl and HV derived from the GenBank database. The tree was generated using the maximum likelihood method with the Kimura 2-parameter substitution model and 1000 bootstrap replicates to assign the confidence level to the branches. The nucleotide sequence obtained in this study is marked in bold.

The oropharyngeal and cloacal swabs tested negative for AI, PMV, *Chlamydia*, WNV, and USUV and positive for HV *DNA polymerase*. Phylogenetic analyses showed that the detected HV sequence was the most closely related to the subfamily *Alphaherpesvirinae* sequences detected in other bird species (Figure 1).

**Figure 2:**
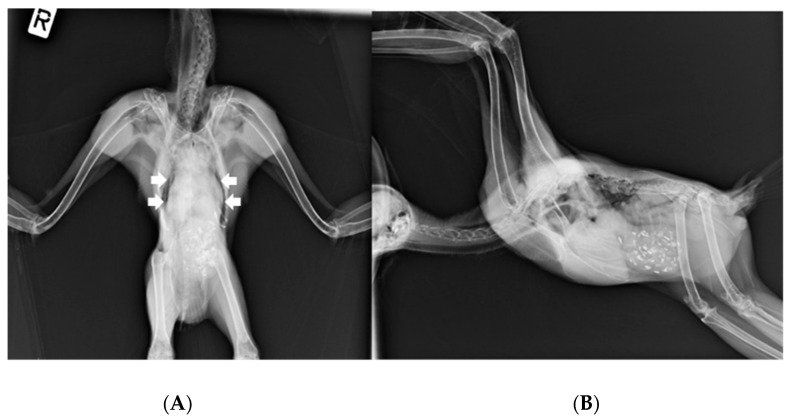
(**A**) Ventrodorsal and (**B**) right lateral radiographic images of the male great grey owl showing widened cardiohepatic waist (marked with white arrows). The “R” mark (image (**A**)) indicates the right side of the body.

On 15 March, the owl’s weight was 1115 g. Ventrodorsal and right lateral radiographic images (Figure 2) under general anesthesia (3% isoflurane in a 1.5 L flow of oxygen with a facemask) revealed a widened cardiohepatic waist, and oropharyngeal swabs were positive for HV presence. Subcutaneous Ringer’s solution and vitamins A, D_3_, and E (Krka, Novo Mesto, Slovenia) were administered.

In the following 3 months, the owl tested positive for HV presence at 2-week intervals despite immunostimulant (Defendyl-Imunoglukan P4H^®^, Medis, Ljubljana, Slovenia) therapy. In the meantime, because of some reports of ocular lesions associated with HV infection [4,22], a complete ophthalmic examination was performed on 6 April 2021, and it showed no abnormalities. On 27 July, the owl’s weight was 915 g, and the BCS was 2. Under general anesthesia, a whole-body radiograph was repeated, which showed changes identical to those of the previous time, and a blood sample was collected from the ulnar vein for plasma biochemical analysis (VetScan VS2, Avian/Reptilian Profile Plus, Abaxis, Union City, CA, USA). Hyperglycemia (23.2 mmol/L; reference interval 13.6–21), hyperalbuminemia (25 g/L; reference interval 15–24), and hyponatremia (146 mmol/L; reference interval 152–168) were observed. The total protein (41 g/L; reference interval 27–41) was in the upper range of the normal limit. No other abnormalities were present in the plasma biochemistry results. Because of the still-positive HV presence in the owl, antiviral therapy with acyclovir (150 mg/kg q24 h) was performed for 7 days.

On 6 August, the owl tested negative for HV presence, with a weight of 975 g and a BCS of 2.5. For the next 6 weeks, the owl was examined weekly for the presence of HV, but no *DNA polymerase* of HV was detected. On 1 September 2021, a blood sample was collected from the ulnar vein for a complete blood count (CBC). The results of the CBC revealed mild anemia (2.04 × 10^12^/L; reference interval 2.1–3.4) and monocytopenia (2%; reference interval 5–23%). The hemoglobin level was 11.6 g/dL, but no reference interval is available for great grey owls; however, based on the reference interval (12.9–16.4 g/dL) for the tawny owl (*Strix aluco*), the results showed mild anemia. A blood smear revealed a mild presence of *Haemoproteus* spp. No other abnormalities were present in the CBC results. On 9 September 2021, the weight was 1050 g, BCS = 3, and the great grey owl was moved into an enclosure with a female great grey owl. On 29 September 2021, the owl’s weight had increased to 1120 g, the BCS was 3, and no HV was detected in the oropharyngeal and cloacal swabs. In the following period, the oropharyngeal and cloacal swabs and weight were tested and recorded every 2 months for HV presence, and no HV was detected. Biochemical analyses on January 6, 2022, revealed hyperalbuminemia (32 g/L; reference interval 15–24) and hypoglobulinemia (8 g/L; reference interval 10–26 g/L). The total protein (41 g/L; reference interval 27–41) was in the upper range of normal. No other abnormalities were present in the plasma biochemistry results. On 21 February 2022, no HV was detected in the oropharyngeal and cloacal swabs, and the owl was revaccinated against WNV. On 28 April, the owl’s weight was 1048 g, BCS = 4/5, and clinical examination showed no abnormalities; however, the owl tested positive again for HV *DNA polymerase* presence. On 20 June 2022, the owl’s weight dropped to 956 g, BCS = 3, and swabs were still positive for HV *DNA polymerase* presence. On 19 July, the bodyweight was 986 g, and the oropharyngeal and cloacal swabs tested positive for HV *DNA polymerase*. The owl was moved to an enclosure, and antiviral therapy with acyclovir (150 mg/kg q24 h) for 7 days was repeated. Owls that were in direct contact (one great grey owl) or that were at the zoo (two Eurasian eagle owls and two snowy owls) were tested, and no HV *DNA polymerase* was detected. On August 1, no HV *DNA polymerase* was detected in the oropharyngeal and cloacal swabs of the great grey owl. For the next 3 weeks, swabs were tested weekly for HV presence, and no HV *DNA polymerase* was detected. Unexpectedly, on 29 August, the owl suddenly died without any clinical signs of illness.

**Figure 3:**
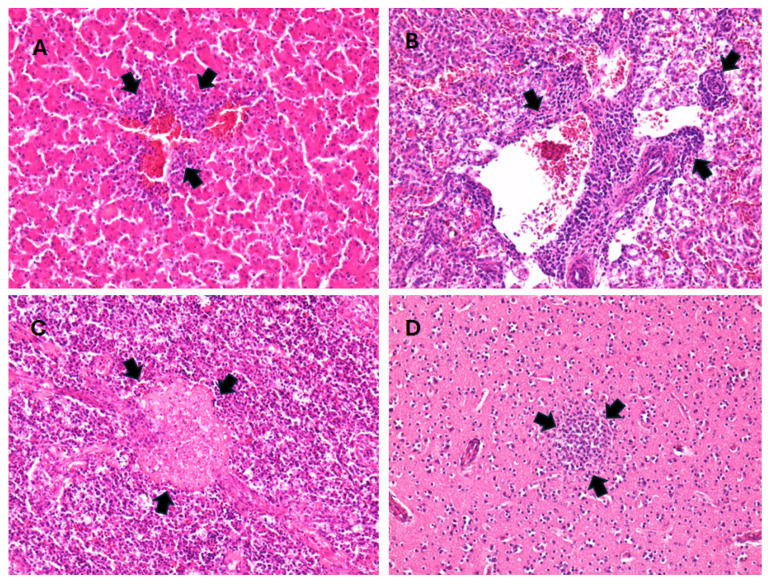
Histopathological lesions: (**A**) liver with mixed cell perivascular infiltrate (marked with black arrows), H&E, 200×; (**B**) kidney with mixed cell perivascular infiltrate (marked with black arrows), H&E, 200×; (**C**) spleen with focal coagulative necrosis (marked with black arrows), H&E, 200×; (**D**) cerebellum with focal gliosis (marked with black arrows), H&E, 200×.

Gross lesions in the great grey owl included an opaque pericardium, a small amount of watery fluid, anemia of the spleen, congestion of the meninges, hemorrhages in the brain, mild congestion of the pancreas, and enteritis. Samples of the heart, lungs, liver, spleen, kidneys, brain, trachea, testes, proventriculus, ventriculus, duodenum, colon, and cecum were collected for histopathological examination. The samples were fixed in 10% buffered formalin and routinely embedded in paraffin. Four micrometer-thick tissue sections were stained with hematoxylin and eosin (H&E) and examined using light microscopy. Routine histopathological analyses were performed at the Institute of Pathology, Wild Animals, Fish, and Bees Veterinary Faculty. Histological findings included moderate multifocal endocarditis, epicarditis, hepatitis, nephritis, and splenitis, with mixed cells, including lymphocytes, macrophages, plasma cells, and heterophils. There were also small multifocal necrosis and lymphoid depletion in the spleen, multifocal gliosis of the cerebrum and cerebellum, lymphoplasmacytic proventriculitis, perivasculitis with mixed cells in the submucosa and muscular layer of the gizzard, from mild to moderate lymphocytic enteritis and typhlocolitis, and multifocal hemorrhages in the gizzard mucosa. Metaplastic ossification was found in the tracheal cartilage, while no lesions were found in the testes (Figure 3).

Bacteriological and parasitological investigations were performed at the Institute of Microbiology and Parasitology’s Veterinary Faculty. Bacteriological examination showed the presence of *Enterococcus faecalis* in the liver sample; *Clostridium perfringens*, *Enterococcus gallinarum*, *Escherichia coli*, and *Enterococcus faecalis* in intestinal samples; and *Enterococcus faecalis* in brain samples. Bacteriological investigation ruled out the presence of *Listeria* spp. from the brain samples. Parasitology examination of the intestinal contents showed the presence of acanthocephalans of the genus *Centrorhynchus*.

An oropharyngeal swab, a cloacal swab, and individual swabs of homogenized liver, kidney, spleen, brain, and lung plus trachea were individually vortexed in 2 mL of phosphate-buffered saline. The total DNA and RNA were extracted from 140 µL of the supernatant with the QIAamp Viral RNA Mini Kit (Qiagen, Hilden, Germany) according to the manufacturer’s instructions.

**Figure 4:**
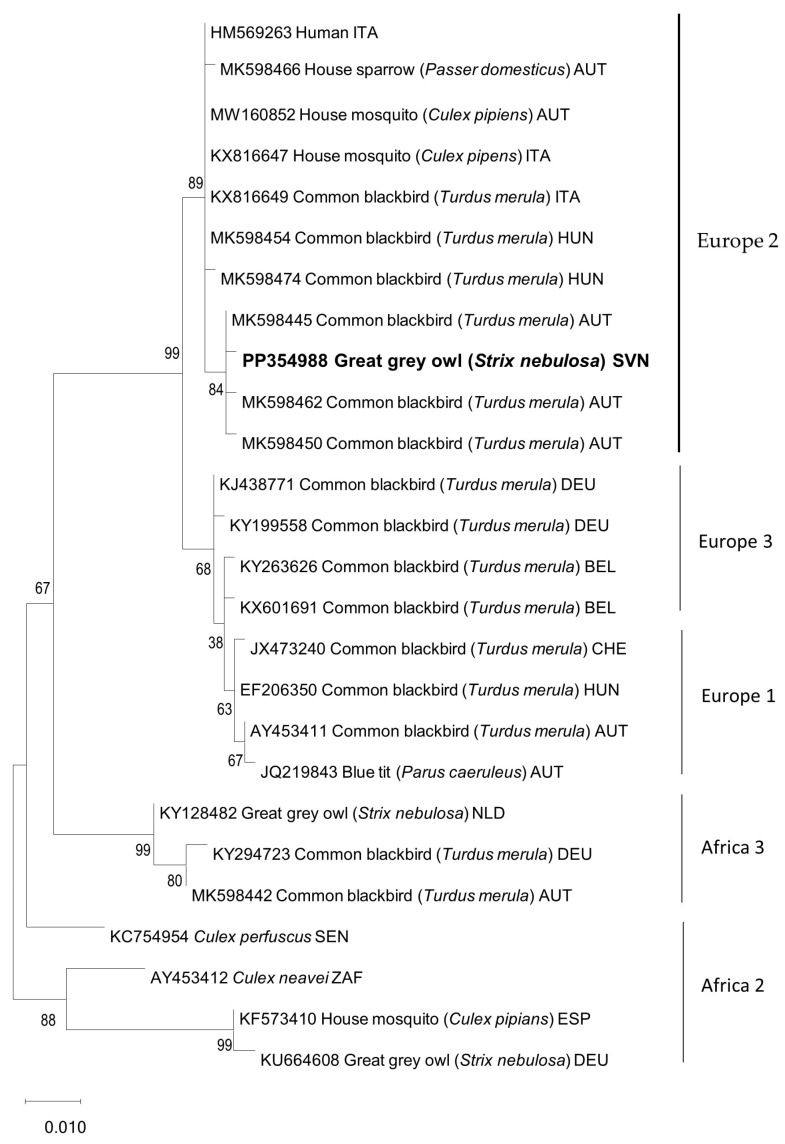
Maximum likelihood phylogeny of partial NS5 protein gene USUV sequences. The phylogenetic tree was constructed using a subset of published USUV sequences from NCBI GenBank, representing USUV strains from lineages Africa 2–3 and Europe 1–3. The tree was generated using the maximum likelihood method with the Kimura 2-parameter model and 1000 bootstrap replicates to assign confidence levels to the branches. The USUV sequence obtained in this study is marked in bold. ITA: Italy; AUT: Austria; HUN: Hungary; SVN: Slovenia; DEU: Germany; BEL: Belgium; CHE: Switzerland; NLD: the Netherlands; SEN: Senegal; ZAF: South Africa; ESP: Spain.

*Chlamydia* spp., PMV, HV, and AI were excluded using standard diagnostic methods. However, RT-PCRs with universal flavivirus primers resulted in clear PCR amplification products of the expected lengths for all the tested organs. Sequence phylogenetic analysis confirmed USUV in the great grey owl and showed clustering within the Europe 2 lineage (Figure 4), with 99.8% nucleotide identical to the most closely related USUV sequence detected in Eurasian blackbirds in Austria.

## 3. Discussion

The great grey owl, despite a 6-month period of HV detection in oropharyngeal and cloacal swabs, showed no clinical signs of disease, nor did histopathological findings show any changes that could possibly be related to HV infection, such as intranuclear eosinophilic inclusion bodies in hepatocytes and necrosis of the liver and spleen. Ventrodorsal radiographic images revealed a widened cardiohepatic waist, which could indicate hepatomegaly due to herpesvirus infection. However, it should be emphasized that the general rule an “hourglass”-shaped junction between the heart and liver does not apply to raptors, in which a commonly much wider cardiohepatic waist and poorly defined junction between the heart and liver are observed. Other possible reasons for the extension of the lateral edges of the liver could be proventricular enlargement (due to disease or due to a full GI tract) or a mass in the coelomic cavity [23]. The phylogenetic analysis of the HV *DNA polymerase* sequence detected in the great grey owl showed a high similarity (99%) with the HV *DNA polymerase* sequence detected in a free-living Ural owl found dead [11], an injured long-eared owl [24], and a live free-living Ural owl, which showed no clinical signs of illness or productivity (i.e., clutch or brood size) deviances [5] (Figure 1). To the best of our knowledge, this is the first report of HV presence in a great grey owl, so the exact impact on this species is unknown; however, based on no recorded clinical sign of illness in this study and the high similarity with the sequence detected in a closely related clinically healthy Ural owl, we could assume that the detected HV has little or no impact on the great grey owl. Nevertheless, such an HV-infected great grey owl represents a possible source of infection for other owls and birds, especially in shared and/or smaller places, such as zoos. HVs are not always restricted to a specific host or tissue, and crossing host or tissue barriers can considerably alter the pathogenicity of the virus [3]. In consideration of this, antiviral therapy with acyclovir was used to reduce the likelihood of HV transmission and potential pathogenicity in other birds. Acyclovir is the first-line treatment for herpes simplex (HSV-1, HSV-2, and VZV) in humans [25]. In birds, some effectiveness has been shown in treatment and prophylaxis for Pacheco’s disease; however, limited data are available for the dosage and use of acyclovir in HV treatment in birds. The treatment/prophylaxis dose depends on the causative agent and on the species of the bird being treated [26,27].

To reduce the chance of the possible side effect of vomiting, a lower dosage of acyclovir than recommended was used in the great grey owl: 150 mg/kg PO q24 h for 7 days [27]. No HV *DNA polymerase* was detected in swabs at the next samplings, which occurred 3 days after the first completed therapy and 6 days after the second completed therapy. The owl tested negative for up to 8 months and 3 weeks after the first and second therapies were completed, respectively, and no side effects were observed. Despite the lower-than-recommended dose, the antivirus treatment successfully stopped the shedding of the virus and, thereby, reduced the transmission and infection possibility to other birds and animals at the zoo. Notably, acyclovir, as an inhibitor targeting *DNA polymerase*/*thymidine kinase*, neither cures the infection nor inhibits viruses in latent phases—it only reduces or stops the replication of the viral genome [25]. Therefore, the reactivation and shedding of the HV weeks/months after the completed therapy are not surprising. Because the owl was not tested for HV presence before the escape from the aviary, the source of the HV infection in this case remains unknown; however, in general, it is assumed that most natural HV infections are acquired during early life, and many healthy birds could remain viremic or persistently infected for prolonged periods of time. It is known that stress factors—in the case of the great grey owl, the escape from the aviary enclosure, remaining in the area outside the zoo, the occasional handling of the owl, and vaccinations—could alter the host–viral balance and lead to the detection of HV in swab samples or even to the disease itself. However, the likelihood regarding HV infection during the weeks that it had escaped should be considered. A study from 2021 has shown that very different HV sequences are present in the wild bird population. Some detected HVs could be prey-related, whereas others showed a tendency to be order- or species-specific [24]. Nevertheless, the possibility that the great grey owl became infected in the time period outside the aviary (zoo) by predating other birds should be emphasized. Last but not least, in addition to the described fatal disease [9,10], ulcerative superficial keratitis, proliferative conjunctivitis, and iris pigmentary changes were described in a great horned owl infected with Strigid herpesvirus-1 (StHV-1) in the United States [4]. In our case, the complete ophthalmic examination of the great grey owl from the Ljubljana Zoo showed no ophthalmic changes. However, it should be emphasized that there was a low (91.2%) nucleotide identity between the HV detected in the great grey owl and the StHV-1 detected in the great horned owl, and it could be that different HVs have different tendencies in target organs and, consequently, exhibit different signs of HV infection. Interestingly, no HV was detected in the female great grey owl despite the fact that she was in direct or close contact through cloacal and oropharyngeal HV shedding by the male great grey owl. However, HV-infected birds testing negative remains a possibility because the latent nonproductive stage of HV in live wild birds is difficult to detect because of the challenging sampling of the possible latency sites of the HV [24]. Considering this, the opposite possibility remains that the female great grey owl and other co-housed owls at the zoo (as silent carriers) could represent the source of the HV infection of the male great grey owl.

In birds, fatal infection with USUV mostly occurs in blackbirds (*Turdus merula*) and house sparrows (*Passer domesticus*) in the order Passeriformes and in great grey owls in the order Strigiformes [14,16,28]. The amplifying host of the USUV is migratory birds, and the main route of transmission is through mosquito bites. These are features shared with WNV and are a reason that the cocirculation of USUV and WNV in various countries in Europe and in some species of birds, as well as in horses, has been described. To the best of our knowledge, this is the first report of USUV in a great grey owl in Slovenia; heretofore, the evidence for USUV activity in Slovenia has been based on the detection of USUV in a common blackbird and a song thrush and on specific antibody detection in selected zoo animals [29,30]. In the case described herein, no clinical signs of illness were noticed despite the fact that great grey owls are one of the most susceptible bird species, with different clinical signs of USUV infection having been described [12,28]. Moreover, no clinical signs of illness due to USUV or other infections were noticed in the female great grey owl held at the same aviary, nor was any clinical deviation noticed in other owls at the zoo, that is, Eurasian eagle owls and snowy owls. Only the HV-positive great grey owl suddenly died because of USUV infection. HV is known to result in stress-induced diseases in birds, meaning that internal or environmental factors could alter the host–virus balance and lead to the disease. One of these factors could be the detected parasites (*Haemoproteus* spp. and *Acanthocephala* spp.); however, in many cases, these factors remain undetermined. Such potentially “immunosuppressed” birds and with a known history of persistent infection with HV could be more susceptible to other viral or bacterial infections and might contribute to a severe or peracute form of USUV infection or, to some extent, at least facilitate these infections. A serological study [30] in 2021 performed in selected animals at the same zoo showed asymptomatic but serologically USUV-positive owls. At the time of that study, great grey owls were not yet present at the zoo, so they were not included in the study. It can be assumed that the serologically positive owls (two Eurasian eagle owls and a snowy owl) at the zoo came into contact with USUV in 2022, but because of serological protection, they did not respond clinically. However, to verify this hypothesis, the serum samples of the owls at the zoo should be rechecked for USUV-specific antibodies, including the surviving female great grey owl that was housed with the diseased male great grey owl. Interestingly, despite the described cross-reactive neutralizing response to USUV after two doses of the WNV vaccine in mice [31], it seems that in the case of the great grey owl, vaccination against WNV did not provide satisfactory protection against USUV. The pathological and histological results showed non-suppurative inflammation in several tissues and are in agreement with other studies [12,21]. USUV was detected in various tissue samples. Phylogenetic analysis showed that the detected USUV sequence obtained in this study clusters together with other Europe 2 sequences detected in neighboring countries (Austria, Hungary, and Italy). These results are not surprising because the Europe 2 lineage is the most detected USUV lineage in European countries [32]. The source of origin of the USUV in the great grey owl is unclear; however, in principle, infected migratory birds and mosquitoes play a crucial role in spreading USUV.

The blood smear of the great grey owl revealed the presence of *Haemoproteus* spp. In most cases, findings of *Haemoproteus* in avian species are related to chronic infection until the end of the host’s lifespan, with the rare presence of clinical disease [33,34]. However, in some cases, severe clinical signs, such as anorexia, lethargy, ataxia, vomiting, or pallor of the skin and mucosal membranes, as well as sudden death, have been reported [35,36,37,38,39,40]. Our case report shows that the *Haemoproteus*-positive great grey owl did not show any clinical deviations despite the fact that there are some reports associated with fatal *Haemoproteus* infections in a snowy owl and a great grey owl [37,41,42]. However, the mild anemia detected by the blood test could be related, among other things, to a *Haemoproteus* infection. *Haemoproteus* and USUV are both vector-transmitted agents; however, USUV is spread by mosquitoes, and *Haemoproteus* is spread by the hippoboscid louse fly. Arthropod vectors are not required for bird-to-bird HV transmission.

## 4. Conclusions

To the best of our knowledge, this is the first documented case of HV presence consequently treated with acyclovir in a great grey owl. This study shows the potential of therapy in the prevention and treatment of HV shedding in great grey owls. Furthermore, this lowers the possibility for spreading the HV to other owls and probably prevents the clinical disease in infected birds. Despite the fact that in the past, HV infection in owls was commonly described with various clinical signs of illness and with a fatal impact on owls, our study did not reveal any potential impact on the clinical health status of the great grey owl that was evaluated. Similarly, no clinical signs of illness were observed before the sudden death of the great grey owl, which occurred because of the USUV infection. Phylogenetic analysis demonstrated the presence of the Europe 2 USUV lineage in the great grey owl.

## Data Availability

The sequence data obtained in this study were deposited in the NCBI database under accession numbers OR239765 (HV) and PP354988 (USUV).
